# The Influence of Argon Cluster Ion Bombardment on the Characteristics of AlN Films on Glass-Ceramics and Si Substrates

**DOI:** 10.3390/nano12040670

**Published:** 2022-02-17

**Authors:** Ivan V. Nikolaev, Pavel V. Geydt, Nikolay G. Korobeishchikov, Aleksandr V. Kapishnikov, Vladimir A. Volodin, Ivan A. Azarov, Vladimir I. Strunin, Evgeny Y. Gerasimov

**Affiliations:** 1 Laboratory of Functional Diagnostics of Low-Dimensional Structures for Nanoelectronics, Novosibirsk State University, 630090 Novosibirsk, Russia; a.kapishnikov@g.nsu.ru (A.V.K.); v.volodin@g.nsu.ru (V.A.V.); i.azarov@nsu.ru (I.A.A.); 2 Department of Applied Physics, Novosibirsk State University, 630090 Novosibirsk, Russia; korobei@nsu.ru; 3 Boreskov Institute of Catalysis, Russian Academy of Sciences (Siberian Branch), 630090 Novosibirsk, Russia; gerasimov@catalysis.ru; 4 Rzhanov Institute of Semiconductor Physics, Russian Academy of Sciences (Siberian Branch), 630090 Novosibirsk, Russia; 5 Department of Experimental Physics and Radiophysics, Dostoevsky Omsk State University, 644077 Omsk, Russia; struninvi@omsu.ru; 6 Institute of Radiophysics and Physical Electronics, Omsk Scientific Center, Russian Academy of Sciences (Siberian Branch), 644024 Omsk, Russia

**Keywords:** aluminum nitride, thin films, gas cluster ion beam, surface smoothing, material characterization

## Abstract

In this paper, the influence of surface modification on the characteristics and properties of AlN thin films on Si and glass-ceramics substrates is investigated. The surface modification was made at various parameters of argon cluster ions. By using XRD and Raman spectroscopy, it was shown that the obtained AlN films have a hexagonal structure with a characteristic direction of texturing along the *c* axis and slight deviations from it. A comparison of the AlN surface morphology obtained by atomic force microscopy before and after cluster processing was demonstrated. This demonstrated that the cluster ions with low energy per atom (E/N = 10 eV/atom) have a high efficiency of surface smoothing. A decrease in the intensity of the Raman peaks and an increase in their full-width after bombardment with cluster ions were found, which may be caused by a change in the physicochemical state of the surface. The optical properties, the quality of the boundaries, and the distribution map of the thickness of the functional layer of AlN were investigated by the methods of spectral and spatial resolution ellipsometry. By using the cross-sectional SEM, the direction of crystallite texturing was demonstrated. The influence of argon cluster ion bombardment on the stoichiometry of samples was analyzed by EDX spectroscopy. The results obtained demonstrate the efficiency of the cluster ion smoothing of polycrystalline thin films for microelectronics, particularly when creating surface acoustic wave resonators.

## 1. Introduction

Aluminum nitride (AlN) possesses useful mechanical (elastic modulus—320–330 GPa; hardness 1100 kg/mm^2^), electrophysical (breakdown field—1.2–1.8 × 10^6^ V/cm; mobility of electrons/holes—135/14 cm^2^/V·s; large energy band gap—6.13–6.23 eV; high resistivity—10^15^ Ω·cm), and thermochemical properties (high thermal conductivity—140–180 W/m·K; coefficient of thermal expansion—4.2–5.3 × 10^−6^ °C^−1^; high melting point—~2200 °C) that enable its use as a material for various electronic devices: acoustic resonators (with the high surface acoustic wave velocity of 12,000 m/s), piezoelectric elements, insulators with a high dielectric constant, and a microelectromechanical system (MEMS) [1,2,3,4,5,6,7,8]. Due to its piezoelectric and pyroelectric parameters, such as the piezoelectric coefficients (e_15_ = −0.33 ~ −0.48 C/m^2^, e_31_ = −0.38 ~ −0.82 C/m^2^, e_33_ = 1.26–2.1 C/m^2^ and d_33_ = 4–6 pC/N) and the relative permittivity coefficient (ε_11_ = ε_22_ = 9, ε_33_ = 11), aluminum nitride is used in optomechanical and electroacoustic devices, phonon crystals, and nonlinear optics [8,9,10,11,12,13,14,15]. As a rule, such devices are in the form of thin-film composite layers containing an AlN layer.

Aluminum nitride films can be prepared by a wide range of methods [16,17,18,19,20,21], which notably include by magnetron sputtering technique [19,20,21]. The use of magnetron sputtering technology can impart the single and composite materials’ electromagnetic shielding, electrical conductivity, and anti-ultraviolet properties, among others. The main advantages of magnetron sputtering are the fast speed of layer growth and the low price of production, which make it a relatively affordable technological method and have led to the widespread use of this technique, including the manufacturing scale [22,23,24].

The resulting films of aluminum nitride can have a different structure and microstructure [25,26,27,28,29], which, in turn, determine their useful properties. In particular, their use in bulk acoustic wave resonators (BAW resonators) presupposes the presence of a c-oriented texture in the material to increase the wave propagation speed [29]. At the same time, morphological properties of the surface, such as roughness, significantly affect the efficiency of its propagation, in the case of surface acoustic wave (SAW) resonators [30,31,32]. To create one or another, or a combined type of resonator, the effective control of bulk and surface properties is required.

Cluster ions can be used to effectively modify the surfaces of various materials due to the collective interaction of hundreds or thousands of weakly bound cluster atoms with target atoms [33]. With the help of cluster ion processing, it is possible to etch and smooth the surface to a subnanometer roughness level [34,35,36,37,38,39,40,41,42,43], while cluster ions, as a rule, damage the subsurface layer with a depth of only a few nanometers [36], without changing the bulk structure of the material. To the best of our knowledge, the change in the morphology of AlN films after treatment with gas cluster ions has not previously been investigated. Generally, only the change in the topography of materials after treatment with clusters is studied [33,34,38,39,40,41].

Previously, we investigated the influence of cluster ion bombardment on the topography of AlN films on the glass-ceramics substrate in the different treatment modes [42]. In our work [42], we showed that cluster ions effectively decrease the surface roughness of an aluminum nitride film to the subnanometer level at etching depths of only tens of nanometers. This work aimed to comprehensively study the effect of cluster treatment on the surface morphology and structure of samples of polycrystalline thin films of AlN deposited on various substrates. Mutually complementary methods such as XRD, AFM, Raman spectroscopy, ellipsometry, and SEM with EDX spectroscopy were used to reveal the features of changes in the surface morphology and characteristics of aluminum nitride films under the minimally invasive impact of the low-energy gas cluster ions.

## 2. Materials and Methods

### 2.1. Deposition Technique and Cluster Treatment Setup

AlN films were obtained by the magnetron sputtering of a target of pure Al (99.99%) in a nitrogen–argon medium (with high-purity gases, 99.999%) at a constant substrate temperature of 350 °C. The substrates were made of monocrystalline Si (100), grade KDB-10 (Ostec-Integra Ltd., Moscow, Russia), and glass ceramics, grade ST-50-1-1-0.6 (C-Component, Moscow, Russia). To improve the adhesion of the AlN films, aluminum and vanadium buffer layers with a thickness of several tens of nanometers were deposited onto the substrates. Additionally, aluminum is important for practical applications because it is used as the electrode and the first structural component of the Bragg reflector [1,4,10]. Before the deposition of the AlN film, the surface of the substrates was cleaned with an alcohol–acetone mixture followed by rinsing with deionized water. The flow rates of the working gases Ar and N_2_ were 4 and 10 sccm, respectively, and the power of the magnetron was 700 W. The background pressure in the vacuum chamber was 0.07 Pa. These parameters were chosen as optimal for AlN film deposition based on the previous set of our experiments. The deposition time was 118 min.

The obtained samples were processed by an unseparated argon cluster ion beam on the CLIUS experimental stand, a brief description of which is presented in [43]. We previously showed that cluster ions with high energy per atom in a cluster E/N = 105 eV/atom intensively sputter the material, and at such energies, the sputtering yield per atom in a cluster is approximately 1, i.e., every 1000 high-energy cluster atoms eject 1000 target atoms [44]. At an energy E/N of several eV/atom in a cluster, the bulk of the initial kinetic energy E is carried away by scattered cluster atoms, and the sputtering yield decreases by almost three orders of magnitude (to 0.001), i.e., only 1 target atom is ejected for every 1000 low-energy cluster atoms [45]. Based on our results presented in [42,44,45], the low-energy regime (E/N = 10 eV/atom, where the kinetic energy of clusters E = 10 keV and their average size N = 1000 atom/cluster) was chosen for processing the AlN thin films. The average beam current density was ≈0.45 μA/cm^2^, and the dose of irradiation with argon cluster ions was ≈2.75 × 10^16^ cm^−2^. The size of the treated area was 3 mm × 5 mm.

### 2.2. Characterization Methods

The obtained thin films were analyzed by X-ray diffraction (XRD) using an ARL X’tra diffractometer (Thermo Fisher Scientific, Basel, Switzerland) with Cu-Kα radiation (λ = 1.5418 Å). Measurements of the thin film samples were performed using asymmetric reflection geometry in the angle range of 2*θ* = 30–85°, the angle between the sample surface and the X-ray beam was 0.7°. The step size during the survey was 0.05°, and the accumulation time was 5 s at each point. The processing of diffraction patterns was carried out by Rietveld refining the full profile in the GSAS–II program [46]. The instrumental broadening of the device was taken into account using a reference sample α-Al_2_O_3_.

The analysis of the morphology of the AlN surface before and after treatment was carried out using an NTEGRA Prima HD atomic force microscope (AFM) (NT–MDT, Zelenograd, Russia). For scanning in the contact mode, ETALON HA_C probes (NT–MDT, Zelenograd, Russia) were used. The radius of the curvature of the tip of the probe is less than 10 nm. The scanned area size varied from 2 μm × 2 μm to 40 μm × 40 μm with a spatial resolution of 1024 × 1024 pixels and a scanning frequency of 1 Hz. Additionally, we scanned areas with a size of 100 μm × 100 μm with a lower resolution (512 × 512 pixels) to take into account the contribution of larger irregularities to roughness. To estimate the etching depth, a mask was used that covered part of the sample surface. After processing with AFM, four areas were scanned at the border of the treated area with a size of 100 μm × 100 μm. The etching depth was estimated from the height difference at the boundary.

To record the Raman spectra (RS), we used a T64000 spectrometer (Horiba Jobin Yvon, Longjumeau, France) using a solid-state laser with a wavelength of *λ* = 514.5 nm. The spectrometer resolution was at worst 2 cm^−1^. The Raman spectrometer was equipped with a confocal microscope with the option of automatically mapping the spectra in the plane with a lateral resolution of 0.8 μm and manually performing in-depth mapping with a resolution of 5 μm. The accumulation time was 60 s; 6 accumulations were made for each spectrum. In each area, 2 Raman spectra were measured, which had good reproducibility. To determine the parameters and ratio of the peaks, the deconvolution of the spectra was carried out using the Fityk program [47]. 

Ellipsometric measurements were carried out on equipment developed at the Institute of Semiconductor Physics SB RAS [48]: spectral ellipsometer “ASEB–5” with a wavelength range of 250–1000 nm and a scanning ellipsometer of high spatial resolution “SCAN–150” with a wavelength *λ* = 633 nm. The size of the probing radiation beams is 1 mm for a spectral ellipsometer and 3 μm for scanning with spatial resolution. The limiting spatial resolution of “SCAN–150”, limited by the precision of the microscrews of the movable table of the ellipsometer, is 5 μm, and the size of the scanning area is up to 150 mm × 150 mm.

**Figure 1:**
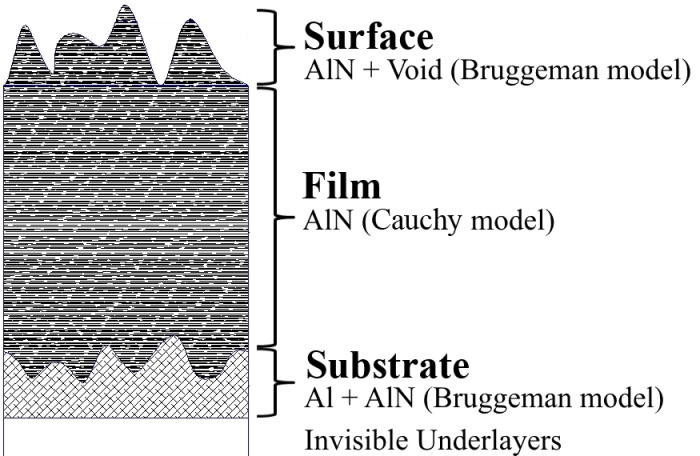
Principal scheme of an ellipsometry multilayer model.

To characterize the samples, a multilayer model was used (Figure 1) with the calculation according to the generalized Fresnel formulas [49]. In our case, we limited the model to three layers: (1) a rough AlN layer on the surface; (2) AlN film under a rough layer; (3) a rough Al buffer layer, which served as a substrate in the ellipsometry model. Aluminum has a high absorption coefficient, and at a thickness of several tens of nanometers, is already opaque to probe radiation, so the underlying layers do not contribute to the measured ellipsometric angles.

Rough layers were described by the Bruggeman effective medium model [50]. Formula (1), where *f* is the filling factor, *N*_1_ and *N*_2_ are the complex refractive indices of the components, and *N_eff_* is the refractive index of the composite medium, is as follows:

(1) $$f\;\frac{N_{1}^{2}-N_{eff}^{2}}{N_{1}^{2}+2N_{eff}^{2}}+\left(1-f\right)\;\frac{N_{2}^{2}-N_{eff}^{2}}{N_{2}^{2}+2N_{eff}^{2}}=0$$

When processing the results, the following algorithm was used: according to the initial data, the approximate thickness of the AlN layer, the volume concentration of aluminum in the transition layer at the lower boundary, and the thickness of the upper rough layer were iteratively selected, and the optical constants of the main layer were then reconstructed according to the Cauchy model [50], which were laid in the transition layers in the next step. Thus, for 3–4 iterations, the model satisfactorily described the experimental data, which were confirmed by convergence at angles of incidence of 50°, 60°, and 70°. Based on the discrepancy between the results for the extreme angles, the error of the resulting thicknesses was estimated. 

The filling factor of the upper rough layer and, where indicated, its thickness, were taken from the AFM data. The thickness of the rough layer was estimated as the arithmetic mean difference in heights *d*_2*Ra*_ = 2 × *R_a_*, where *R_a_* is the arithmetic mean surface roughness measured by AFM on a scale of 40 μm × 40 μm; and the average filling volume was estimated from the surface profiles.

Additionally, an experiment was carried out to study the morphology of an AlN sample on a Sitall (glass-ceramics) substrate after treatment with cluster ions on a TESCAN SO-LARIS FE-SEM double-beam scanning electron microscope (SEM) (TESCAN, Brno, Czech Republic) with an accelerating voltage of 30 kV in secondary electron modes. The device is equipped with an AztecLive energy dispersive X-ray characteristic radiation (EDX) spectrometer (Oxford Instruments, Abingdon, United Kingdom) with a semiconductor Si-detector with an energy resolution of 128 eV. To study the morphology of the sample, a lamella was fabricated; etching was carried out in the 20 μm section.

## 3. Results and Discussion

### 3.1. XRD Analysis

**Figure 2:**
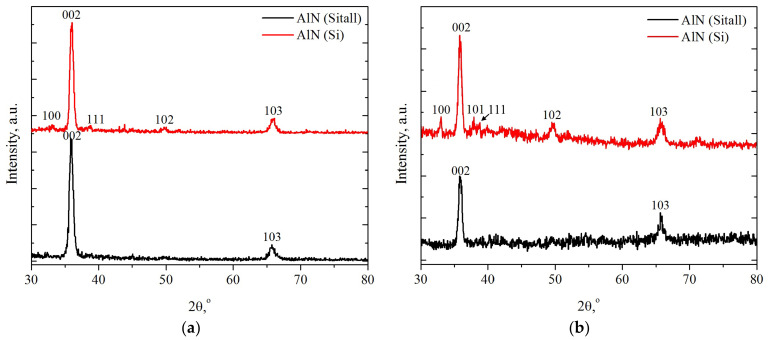
Comparative X-ray diffraction patterns of AlN films on Sitall (glass-ceramics) and Si substrates: (**a**) as-prepared; (**b**) after treatment with argon cluster.

According to XRD data (Figure 2), the AlN obtained in the films has a wurtzite-type structure (space group P63mc, JCPDS no. 25–1133). The presence of clearly pronounced reflections of this phase, such as 002 and 103, as well as less intense 100, 101, and 102, was noted in the diffraction patterns. The presence of a weak 111 reflection (d = 2.334 Å), which belongs to the Al phase (JCPDS no. 4–787), can be explained by partial diffraction from the Al buffer layer.

The diffraction pattern for AlN films indicates the presence in the samples of the preferred orientation of crystallites in the direction of the *c* axis, with the March-Dollars parameter in the range of 0.35–0.43, which is in good agreement with the literature data [25,28,51]. At the same time, the presence of reflections 103, 102, and 101 indicates a deviation of some of the crystallites relative to the growth direction [52], which may be related to the degree of roughness of the aluminum buffer layer and the growth of unoriented crystallites on it [53].

The AlN lattice parameters of all samples are, on average, *a* = 3.115 Å and *c* = 5.000 Å. The calculated values are larger than the reference ones (*a* = 3.1114 Å, *c* = 4.9792 Å), which may indicate the presence of defects in the obtained films. The aluminum nitride phase is sufficiently well crystallized, and the average crystallite size is approximately 100–150 nm. No significant changes in the phase composition of the films after processing were observed on the X-ray diffraction patterns. It should be noted that the intensity of the X-ray diffraction patterns after cluster treatment is lower because of the difference between the initial and treated sample sizes. Possible subsurface amorphization after argon cluster treatment can also make some insignificant background fluctuations. Intensity for other directions, such as 100 or 101, is slightly increased, however, this change may be due to a better-resolved signal from the lowest disordered AlN layer. However, there are no major differences in phase composition between initial and treated samples.

### 3.2. Surface Analysis

**Figure 3:**
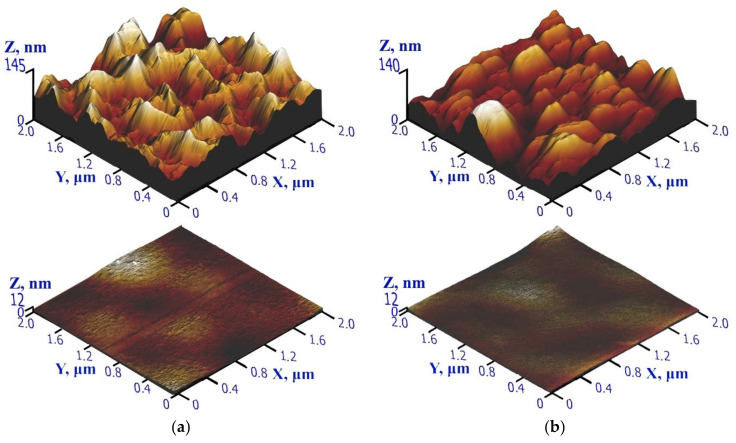
AFM images of AlN surfaces before (**top**) and after (**bottom**) Ar cluster ion beam on Sitall (glass-ceramics) (**a**) and Si (**b**) substrates.

Figure 3 shows the AFM images of the initial surface of a polycrystalline AlN film and surfaces after treatment with cluster ions on different substrates. In this case, the most visual and reflecting surface structures are images with a scale of 2 μm × 2 μm.

During magnetron sputtering, crystallites grow on fine-equiaxed grains located on the surface and with increasing film thickness, acquire a columnar microstructure with an axis perpendicular to the substrate surface [54,55]. On the initial surface of AlN, the tops of columnar formations are observed (Figure 3) with a lateral size in the range of 250–550 nm. Taking into account the XRD data, it can be assumed that each “column” growing perpendicular to the substrate surface is polycrystalline and consists of approximately 3–6 crystallites in a cross-section. As a result of the treatment of the AlN film with low-energy argon cluster ions (*E/N* = 10 eV/atom), the maximum difference of irregularities on the surface *R_t_* decreased to 12 nm, which is approximately 12 times less than the initial value of *R_t_* (Figure 3b).

**Figure 4:**
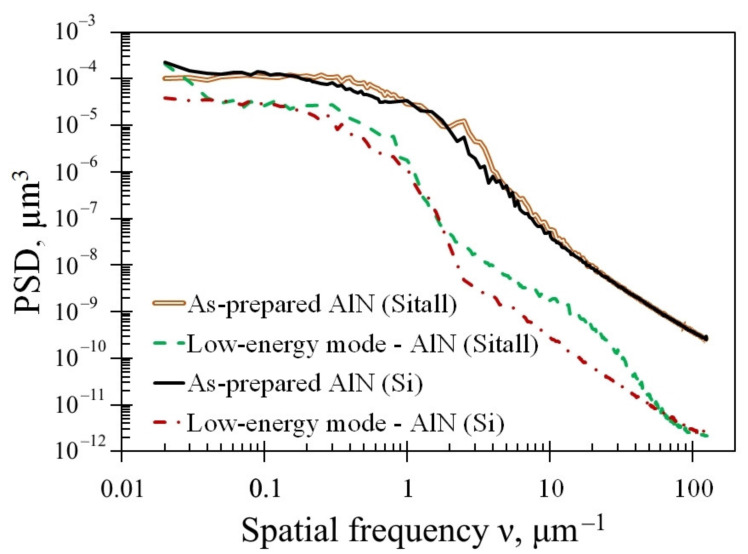
PSD functions of the roughness of the AlN surface before and after cluster ion treatment on the different substrates.

Earlier, in [43,56,57,58,59], it was shown that it is possible to take into account and estimate the contribution of irregularities to the surface roughness depending on their lateral size. The power spectral density (PSD) function of roughness is the fast Fourier transform of a surface elevation gradient dataset [57,58]. This function allows you to take into account the lateral size of irregularities, the value of which is inversely proportional to the spatial frequency of roughness *ν*. The integral of the PSD function is the effective roughness parameter σ*_eff_*, which takes into account the lateral size of the roughness. Scanning different sizes of the scanning area (from 2 μm × 2 μm to 100 μm × 100 μm) allows constructing generalized PSD-functions in a wide range of spatial roughness frequencies *ν* from 0.02 to 125 μm^−1^, which corresponds to the lateral dimensions of irregularities in the range of 8–50 μm [43]. Generalized PSD functions before and after treatment with argon cluster ions are shown in Figure 4.

As can be seen in Figure 4, the roughness decreased after processing the entire measured range of spatial frequencies. It should be noted that aluminum nitride on a silicon substrate is smoothed better in the range of 2–40 μm^−1^, that is, irregularities with a transverse size of 25–500 nm are better smoothed. As a result, at the small scale (2 μm × 2 μm), the AlN roughness on the Si substrate decreased by 12–14 times, while on the Sitall substrate, it decreased by 7.5 times, as can be seen in Table 1. The AlN is smoothed approximately 22% better on the Si substrate than on the glass-ceramic substrate, which can be seen from the difference in the values of the generalized effective roughness ⟨σ*_total_*⟩.

### 3.3. Raman Spectroscopy

**Figure 5:**
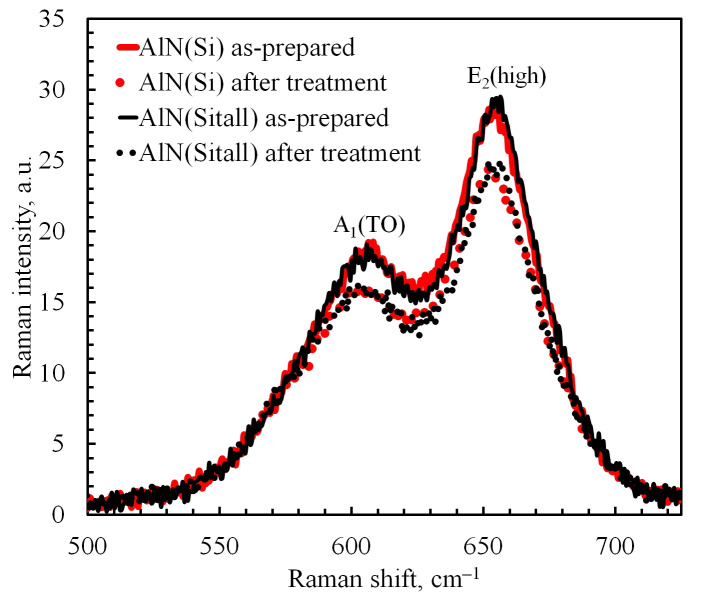
Raman spectra of AlN before (solid) and after (dot) treatment with cluster ions.

AlN has a crystal structure of wurtzite type. It is known from the literature [60,61] that, for AlN, the A_1_(TO) and E_2_(high) modes, located in the spectral range of 600–700 cm^−1^, are most sensitive to the scattering geometry. As the authors of [61] wrote, the vibrational mode E_2_(high) is excited by an electric field perpendicular to the *c* direction, and the A_1_(TO)mode is excited by an electric field parallel to the *c* direction. Thus, in the case of ideal orientation (002), the peak of the E_2_(high), the mode should be observed, and the A_1_(TO) mode should be absent. As can be seen from Figure 5, in our case, both modes are present, which corresponds to the presence of grains with a different orientation (different from 002), the electric field of which has components parallel and perpendicular to the *c* axis, which excite both the E_2_(high) mode and the A_1_(TO) mode. Consequently, the enhancement of the A_1_(TO) mode is caused by the appearance of grains of other orientations, which is consistent with XRD data, in which reflections of other orientations are observed.

After treatment with argon cluster ions, the intensity of the Raman peaks is lower than the intensity of the peaks in the original film (Figure 5). The Raman spectra obtained by us correspond to polycrystalline AlN and are similar to the spectra of synthesized AlN nanophase products [62].

The Fityk program [47] was used to decompose the spectrum and obtain the parameters of individual peaks. The band profile was chosen as Pearson VII Function (2) which better describes our spectra than other functions. The data obtained are shown in Table 2:

(2) $$I\left(x\right)=\frac{I_{peak}}{{\left[1+\left(\frac{x-x_{peak}}{HWHM}\right)\left(2^{\frac{1}{shape}}-1\right)\right]}^{shape}}$$

where *I(x)* is the spectrum intensity, *x* is the Raman shift (cm^−1^), *I_peak_* is the peak intensity, *x_peak_* is the Raman shift of peak (cm^−1^), *HWHM* is the half-width at half maximum, and *shape* is the fitting parameter. 

After processing, the positions of the peaks for both modes were insignificantly shifted (0.1–2.4 cm^−1^), and the FWHW increased by 4–5.7%, except for the E_2_(high) mode for AlN (on Sitall), where the change was <1%. The FWHW of the E_2_(high) peaks varied from 38.8 to 41.5 cm^−1^, which is between 3 cm^−1^ (high-quality AlN crystals) and 50 cm^−1^ (highly defective crystals) [61]. The broadening of the Raman peaks may be the result of phonon scattering caused by fine grains, point defects, interfaces, and stress gradients [63]. The insignificant broadening of the peaks could be influenced by the presumptive formation of an amorphous layer with a depth of several nanometers as a result of treatment with cluster ions. It is known that the width of a Raman peak for amorphous material is bigger than the width of a Raman peak for crystalline material. Generally speaking, the shape and width of a Raman peak depend on phonon correlation length. In nanocrystalline materials, the phonon correlation length is determined by the size of the nanocrystals [64]. However, the phonon correlation length can also be determined by the presence of defects (as scattering centers for phonons) and the distance between defects on surface or surface roughness (also scattering centers for phonons). Therefore, an increase in the width of the peaks can be due to the presence of an amorphous phase and the change in the quality of the surface. It should also be noted that the FWHW of the E_2_(high) peaks is less than that of the A_1_(TO) peaks, which may be due to the different crystallite sizes or defect concentrations in different directions.

It was shown in [60] that the ratio of the A_1_(TO)/E_2_(high) peaks correlates with the piezoelectric coefficient d_33_. The larger the ratio A_1_(TO)/E_2_(high), the smaller d_33_ will be registered in the samples. The Raman spectrum obtained in our work best matches the Raman spectrum of sample #3, i.e., polycrystalline AlN, considered in ref. [60]. Redkin and colleagues showed that the ratio A_1_(TO)/E_2_(high) ≈ 0.12 corresponds to the piezoelectric coefficient *d*_33_ = 4–5 pC/N. If we extrapolate the nonlinear dependence from [60] for the results of our work, it turns out that the ratio A_1_(TO)/E_2_(high) ≈ 0.64–0.67 corresponds to the piezoelectric coefficient *d*_33_ ≈ 2 pC/N or less. This means that the sample demonstrates piezoresponse which, despite being smaller than the table value, can be explained by defects in the crystal structure of polycrystalline AlN, imperfections in the stoichiometry of Al:N (observed by SEM EDX), and by small-angle inclinations of the crystal growth axis toward the normal *c* axis observed in the cross-sectional SEM images.

### 3.4. Ellipsometry

**Figure 6:**
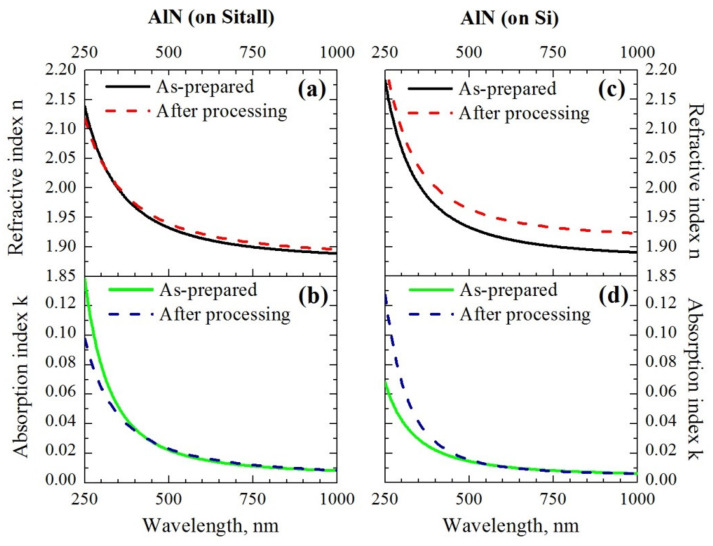
The optical constants dependencies on wavelength *λ* for the AlN layer: (**a**) the refractive index *n* as-prepared and after low-energy mode on the Sitall substrate; (**b**) the absorption index *k* on Sitall; (**c**) the refractive index *n* on Si; and (**d**) the absorption index *k* on Si. Parameter errors: *δn* = 0.01 and *δk* = 0.002.

First of all, measurements were carried out on a spectral ellipsometer. Dispersion curves for AlN before and after treatment with the argon cluster ions on different substrates are shown below (Figure 6), calculated according to the results of the models described in Section 2.2 (Figure S1). For the treated surface, the thickness and filling factor of the rough layer are taken from the AFM data. The absorption index values in most of the range do not exceed a few hundredths, which indicates the good dielectric properties of AlN. The refraction index is close to the literature data [65,66]. Significant changes in the refraction index indirectly confirm the possibility of the effect of clusters on the entire depth of the film.

Table 3 shows the comparative results of ellipsometry and AFM. For the initial AlN films, the estimate of the rough layer thickness is close to the AFM data, which confirms the correctness of the approaches used. For the treated AlN films, the simulation results converged much worse, both according to the estimates of the rough layer thicknesses and to the etching depth obtained from the AFM data. Therefore, a model was applied in which the thickness of the rough layer was set based on the AFM results. With this refinement of the model, the values of the etching depth closer to the AFM estimates were obtained, however, at the same time, the refractive index of the entire AlN layer increased significantly (see Figure 6a,c). The absorption index in the main region of the range (500–1000 nm) practically did not change, and insignificant changes in the short-wavelength region (250–500 nm) are associated, judging from further data, with the inhomogeneity of the thickness of the main AlN layer. Thickness inhomogeneity reduces the amplitude of interference oscillations such as a small addition to the absorption index. Additionally, the discrepancies in the AFM and ellipsometry data can be associated with the formation of an amorphous layer after processing with clusters [36]. Attempts to introduce an additional layer of 20–30 nm thickness resulted in the very low values of the refractive index (*n* = 1.2–1.3) of this layer. Therefore, the issue of changes in the subsurface layer and the thickness of AlN requires further study.

**Figure 7:**
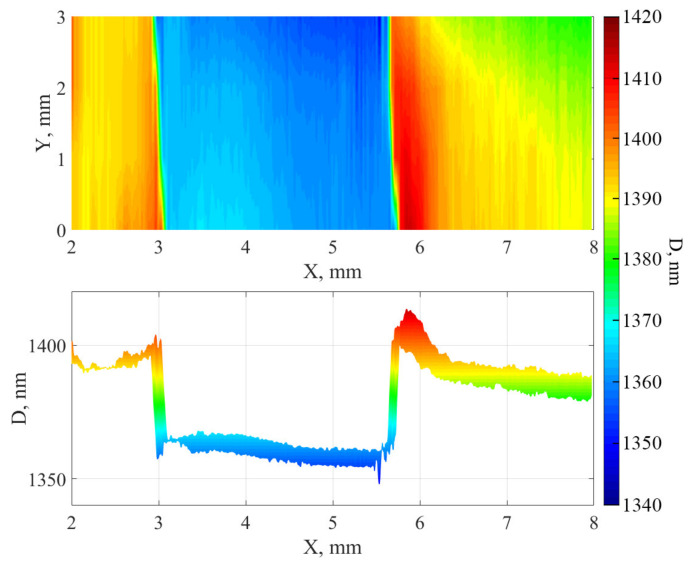
Principal map of the distribution of thicknesses of the aluminum nitride film on Sitall. Top view (**top**) and cross-section of the film (**bottom**).

**Figure 8:**
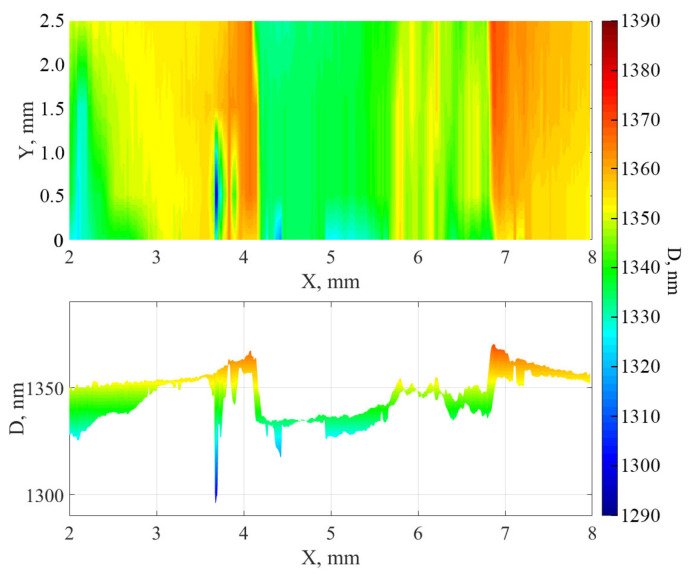
Principal map of the distribution of thicknesses of the aluminum nitride film on Si. Top view (**top**) and cross-section of the film (**bottom**).

The surface was also mapped using a spatial scanning ellipsometer. During processing, a model with a fixed transition layer thickness of 20 nm was used to construct a principal map of the thickness distribution (Figure 7 and Figure 8). As can be seen from the distributions, when the surface is treated with cluster argon ions and a mask is used for the sample, “elongated hillocks” are formed along the edges of the treated area, the cause of which may be the process of redeposition or the significant lateral displacement of atoms at the edges of the area.

These results make it possible to explain the overestimated values of the etching depth in the second and fifth columns of Table 3 according to the AFM data, since AFM scanning was carried out over a smaller area and the size of the “step” was approximately reckoned from the apex of the “elongated hillock”.

### 3.5. Scanning Electron Microscopy

**Figure 9:**
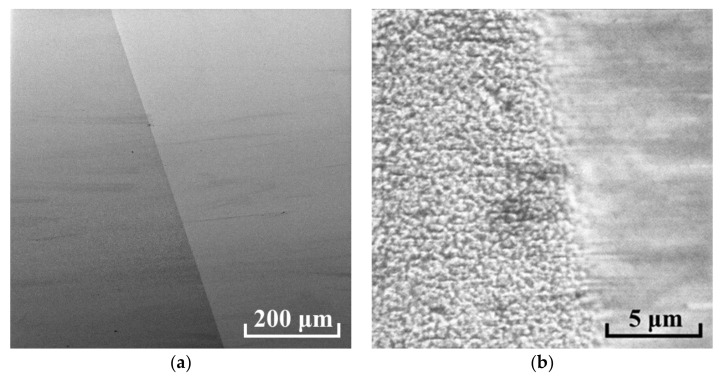
SEM images of the border between as-prepared (left sides) and smoothed areas (right sides) of AlN film on Sitall substrate for different magnifications marked by scale bar: (**a**) 200 µm, (**b**) 5 µm.

An AlN sample on a Sitall substrate was examined by SEM. The sample shows two different zones with different conductivity (Figure 9). At higher magnification, a different surface morphology is visible. In the area of treatment with a cluster ion beam, the surface is smooth; in the neighboring area, roughness is observed with the observation of a characteristic porous structure.

**Figure 10:**
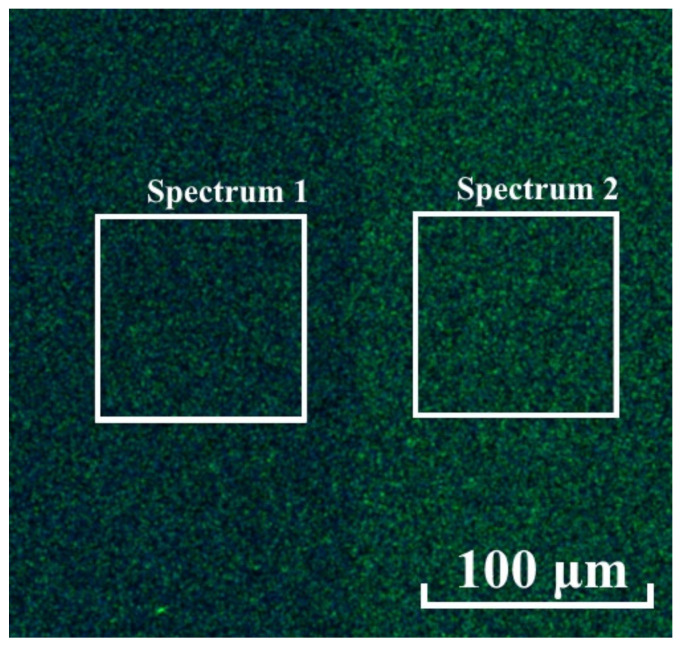
EDX mapping for the cluster ion-treated area (left) and untreated area (right).

Chemical mapping experiments have shown that these areas differ in chemical composition (Figure 10). In the untreated (right) area, the contents of the main elements Al, N, and O were, respectively, 44.2, 40.2, and 3.5%, whilst in the polished (left) area, the contents were, respectively, 43.2, 39.4, 5.8%. It is also worth noting the different carbon contents in these areas, although the sample was wiped with ethyl alcohol before loading into the microscope, which could change the chemical composition. It should be noted that the EDX map was mirrored due to the peculiarities of the experiment.

**Figure 11:**
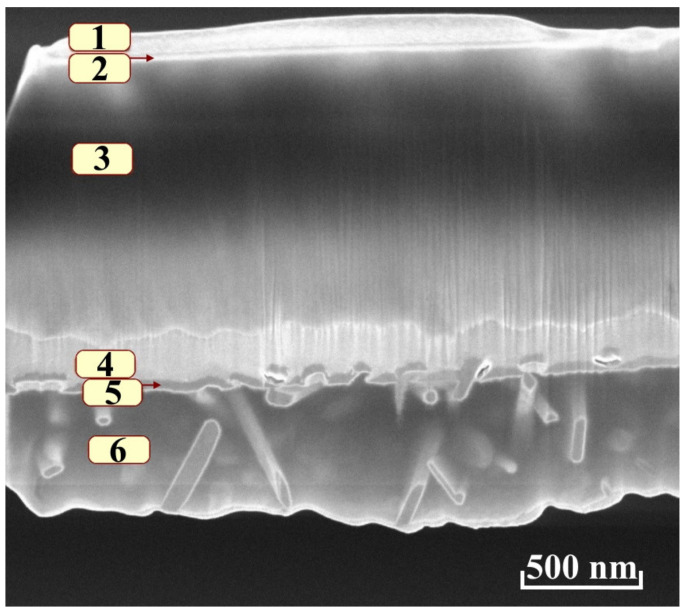
Cross-section of the lamella after thinning: 1—platinum; 2—amorphous layer of AlN; 3—AlN; 4—aluminum; 5—vanadium; and 6—ceramic substrate (Sitall).

Furthermore, a lamella was prepared from the treated area of the sample. On the surface of the lamella, a protective platinum deposition was applied, produced in the column of the microscope. Then, the lamellae were mounted on a grid for electron microscopy and further thinning to a thickness of approximately 100 nm with an ion beam. The general view of the lamella after thinning is shown in Figure 11. This figure shows six layers corresponding to different zones in this sample: layer 1 is the platinum protective coating; layer 2 is the amorphization zone of AlN; layer 3 is the massive AlN film; buffer layer 4 is aluminum; buffer layer 5 is vanadium; layer 6 is Sitall (ceramic substrate, determined by its composition as a Ca–Ti–Si composite). The roughness of the aluminum buffer layer and some deviations of texturing axis are observed on this micrograph.

Summing up, it should be noted that according to the X-ray phase analysis data, it can be argued that the grown AlN films are polycrystalline (crystallite size of the order of 100–150 nm) with a predominant orientation along the *c* axis. At the same time, there are small deviations in the system concerning the texturing axis. The phase composition of the films after treatment with cluster ions did not change significantly. Raman spectra correspond to polycrystalline films with other orientations, which is in good agreement with X-ray phase analysis. After treatment with argon cluster ions, a decrease in the intensity of the peaks and an increase in the half-width of the peaks of the A_1_(TO) and E_2_(high) modes by 4–5.7% are observed, which can be caused by both an increase in the number of film defects and the formation of an amorphous subsurface layer. Ellipsometric studies have shown the good optical quality of AlN films, as confirmed by low values of the absorption index and close to the literary values of dispersion. The interfaces at the AlN boundaries are not sharp; the thickness of the transition layers in the original films is several tens of nanometers at both boundaries, which is confirmed by direct methods, e.g., for the inner boundary with aluminum; and the SEM image of the cross-section was used, while for the outer boundary, the AFM was used. Spatial resolution ellipsometry made it possible to establish a characteristic profile of the surface treated with ${\mathrm{Ar}}_{1000}^{+}$ clusters, on which material displacements from the treated area under the mask are visible. The ellipsometry data for the treated surface show a significant change in the optical properties of AlN, notably an increase in the refractive index, possibly over the entire layer depth of ~1.5 μm, which requires further study.

After treatment with clusters, the roughness of AlN on an Si substrate on small scales (2 μm × 2 μm) decreased two times more than on a Sitall substrate. In this case, the roughness of AlN on a Si substrate, generalized over a wide range of lateral irregularities, is approximately 22% lower, which is explained by the better smoothing of irregularities with a transverse size from 25 to 500 nm. It should be noted that low-energy cluster ions provide effective smoothing of the AlN surface at a small etching depth of the target. The obtained results on the roughness and piezoelectric properties demonstrate the effectiveness of the cluster ion smoothing technique for obtaining smooth surfaces in a minimally invasive way when creating BAW- and SAW-resonators.

## 4. Conclusions

The impact of an effective cluster ion beam on AlN thin films was demonstrated. The structure and optical characteristics of AlN films on glass-ceramics and Si substrates before and after argon cluster ion bombardment were investigated for the first time. The combination of applied diagnostic methods made it possible to comprehensively characterize the subsurface layer of the films before and after smoothing. We observed the stability of the phase composition, the absence of crack formation, and the negligible change in the elemental composition of the subsurface layer of AlN films. The insignificant influence of clusters on the film bulk was indirectly confirmed by detailed XRD analysis, Raman spectroscopy, and ellipsometry.

The achieved low-roughness values of oriented polycrystalline films are satisfactory for the production of microelectronic structures, taking into account the need for the deposition of following layers during the formation of heterostructures, including the production of resonators based on piezoactive AlN. Since roughness harms the spread of acoustic waves in BAW- and SAW-resonators, the smoothness of the surface of the piezoactive AlN films is an extremely important parameter for the devices, which can be achieved with our technique reducing the surface roughness.

## Figures and Tables

**Table 1:** The roughness parameters of AlN thin films.

Scan Size, μm^2^	Roughness Parameter	AlN (on Si)		AlN (on Sitall)	
		As Prepared	After ${\mathrm{Ar}}_{1000}^{+}$ Cluster Processing	As Prepared	After ${\mathrm{Ar}}_{1000}^{+}$ Cluster Processing
100 × 100	⟨*R_q_*⟩*,* nm	9.7	4.2	9.2	4.8
	⟨σ*_eff_*⟩*,* nm	10.0	4.5	9.7	5.0
40 × 40	⟨*R_q_*⟩*,* nm	11.0	4.2	13.7	4.8
	⟨σ*_eff_*⟩*,* nm	11.8	4.2	14.8	5.0
2 × 2	⟨*R_q_*⟩*,* nm	20.5	1.5	22.3	1.8
	⟨σ*_eff_*⟩*,* nm	21.5	1.5	24.0	3.2
-	⟨σ*_total_*⟩*,* nm	29.2	9.7	32.5	12.5

Note: ⟨*R_q_*⟩ is root-mean-square roughness; ⟨*σ_eff_*⟩ is average effective roughness; ⟨σ*_total_*⟩ is effective roughness, generalized over the entire measured range of spatial frequencies *ν.*

**Table 2:** Raman band parameters and A_1_(TO)/E_2_(high) intensity ratios for the AlN films.

Raman Peak Parameters	AlN (on Si)		AlN (on Sitall)	
	As Prepared	After ${\mathrm{Ar}}_{1000}^{+}$ Cluster Processing	As Prepared	After ${\mathrm{Ar}}_{1000}^{+}$ Cluster Processing
A_1_(TO) position, cm^−1^	602.0	599.6	601.0	599.7
A_1_(TO) FWHM, cm^−1^	57.1	59.3	55.6	58.7
E_2_(high) position, cm^−1^	655.9	655.2	655.9	656.0
E_2_(high) FWHM, cm^−1^	39.5	41.5	38.8	39.1
A_1_(TO)/E_2_(high)	0.67	0.64	0.65	0.65

**Table 3:** Ellipsometric and AFM data of AlN thin films.

**Parameter**	AlN (on Sitall)			AlN (on Si)		
	As Prepared	After ${\mathrm{Ar}}_{1000}^{+}$ Cluster Processing	*After ${\mathrm{Ar}}_{1000}^{+}$ Cluster Processing	As Prepared	After ${\mathrm{Ar}}_{1000}^{+}$ Cluster Processing	*After ${\mathrm{Ar}}_{1000}^{+}$ Cluster Processing
*d*_2*Ra*_(AFM), ± 0.05 nm	14–15.6	7.4	7.4	15–22	6.4–7.2	6.4–7.2
*d*_2*Ra*_, ± 1.5 nm	20.2	12.4	7.4	18.7	26.5	7.2
*D_total_*, ± 5 nm	1490	1465	1453	1460	1455	1415
*H_etch_*, ± 5 nm	-	−25	−37	-	−5	−45
*H_etch_*(AFM), ± 5 nm	-	−43	−43	-	−30	−30

Note: *d*_2*Ra*_(AFM) = 2 × *R_a_* is the rough layer thickness estimated according to the AFM data; *d*_2*Ra*_ is the rough layer thickness estimated according to the ellipsometry data; *D_total_* is the AlN film thickness taking into account the rough layer*; H_etch_* and *H_etch_*(AFM) are the etching depth from the ellipsometry and AFM data, respectively; *After ${\mathrm{Ar}}_{1000}^{+}$ is the column of the model results, in which the thickness estimated from the AFM data for the rough layer was used; and the AlN variance was that which was desired.

## Data Availability

Not applicable.

## Supplementary Materials

The following is available online at https://www.mdpi.com/article/10.3390/nano12040670/s1. Figure S1: Results of ellipsometry multilayer model.
