# Supplementary material for: The Influence of Argon Cluster Ion Bombardment on the Characteristics of AlN Films on Glass-Ceramics and Si Substrates
Source: Nanomaterials (Basel). 2022 Feb 17;12(4):670. doi: 10.3390/nano12040670 (PMC8878031; doi:10.3390/nano12040670)
Supplement: Supplementary file 1 [file nanomaterials-12-00670-s001.zip › nanomaterials-1522316-supplementary.pdf]

The Influence of Argon Cluster Ion Bombardment on the Characteristics of AlN films on Glass-Ceramics and Si Substrates

Ivan V. Nikolaev [1,2,*], Pavel V. Geydt [1,*], Nikolay G. Korobeishchikov [2], Aleksandr V. Kapishnikov [1,3], Vladimir A. Volodin [1,4], Ivan A. Azarov [1,4], Vladimir I. Strunin [5,6] and Evgeny Y. Gerasimov [3]

[1] Laboratory of Functional Diagnostics of Low-dimensional Structures for Nanoelectronics, Novosibirsk State University, Novosibirsk 630090, Russia; a.kapishnikov@g.nsu.ru (A.V.K.); v.volodin@g.nsu.ru (V.A.V.); i.azarov@nsu.ru (I.A.A.)

[2] Department of Applied Physics, Novosibirsk State University, Novosibirsk 630090, Russia; korobei@nsu.ru

[3] Boreskov Institute of Catalysis, Russian Academy of Sciences (Siberian Branch), Novosibirsk 630090, Russia; gerasimov@catalysis.ru

[4] Rzhanov Institute of Semiconductor Physics, Russian Academy of Sciences (Siberian Branch), Novosibirsk 630090, Russia

[5] Department of Experimental Physics and Radiophysics, Dostoevsky Omsk State University, Omsk 644077, Russia; struninvi@omsu.ru

[6] Institute of Radiophysics and Physical Electronics, Omsk Scientific Center, Russian Academy of Sciences (Siberian Branch), Omsk 644024, Russia

* Correspondence: i.nikolaev@nsu.ru (I.V.N.); p.geydt@nsu.ru (P.V.G.)

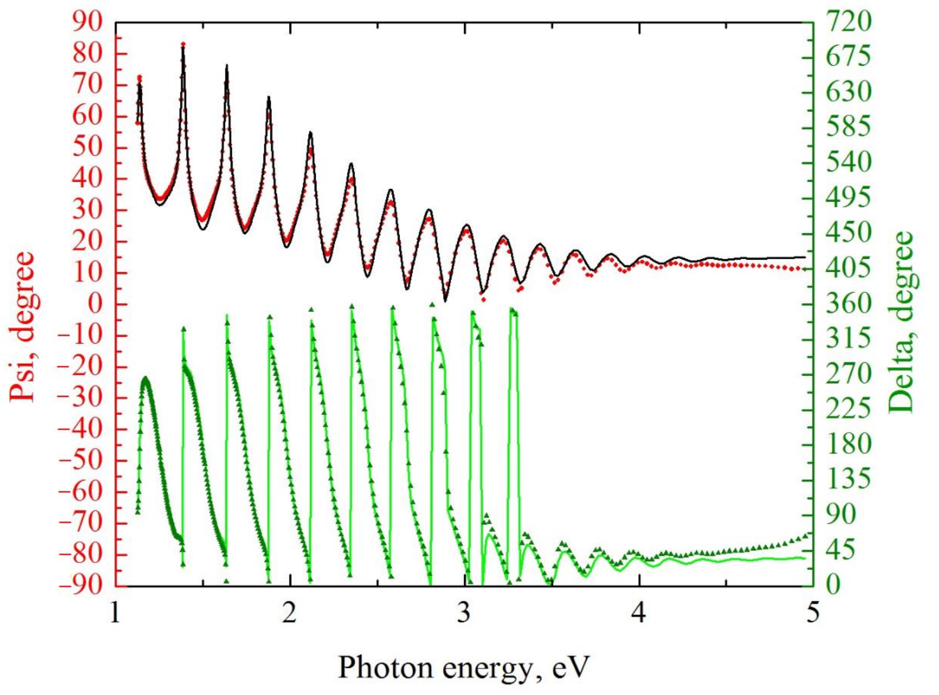

Figure S1. Results of ellipsometry multilayer model.
